# *Escherichia coli* as a Tool for Disease Risk Assessment of Drinking Water Sources

**DOI:** 10.1155/2020/2534130

**Published:** 2020-06-15

**Authors:** Stephen T. Odonkor, Tahiru Mahami

**Affiliations:** ^1^School of Public Service and Governance, Ghana Institute of Management and Public Administration, Accra, Ghana; ^2^Ghana Atomic Energy Commission, Kwabenya, Accra, Ghana

## Abstract

Many diseases have been associated with poor drinking water quality including diseases caused by diarrheagenic pathogens, especially in developing countries where access to a consistent water supply is a problem. The objective of the study was to evaluate the health risks associated with the sources of drinking water in the Dangme West District of Ghana using *E. coli* as a measurement tool, aiming at ascertaining the paths leading to contamination of the water sources. A total of 464 water samples were obtained for analysis. Sampling covered a year across the dry and wet seasons in Ghana. Water samples were obtained from groundwater and surface water sources. *E. coli* counts were determined using the most probable number method (MPN). Disease risk assessment was carried out using the WHO drinking water risk assessment guidelines. Generally, the study revealed significantly higher *E. coli* counts in the wet season than in the dry season. Among the water samples analyzed, surface water, especially from the dams, was found to pose the highest disease risk as compared to the other water sources. Samples from groundwater sources, especially boreholes, posed the lowest disease risk. In conclusion, observations from the study implied that most water sources in the study district are highly polluted with bacteria pathogens beyond recommended safety guidelines. The main causes of faecal contamination in these water sources were purported to be anthropogenic. Therefore, there is a need to formulate a policy aimed at managing and improving rural water sources.

## 1. Introduction

Water is today a scarce resource, particularly in the developing world. Globally, 663 million people do not have access to safe water [1]. Rural communities in sub-Saharan Africa account for more than 50% of those lacking potable water [2, 3]. Most of these communities, therefore, rely on untreated sources such as streams, dams, boreholes, wells, and rivers to meet fundamental needs such as drinking, sanitation, and cooking and for their sustainable development [4]. However, these untreated sources are contributing significantly to the global burden of disease, as a result of water-related infectious diseases such as cholera, dysentery, and typhoid [5]. Every year, millions of lives are lost worldwide with a chunk in developing countries as a result of waterborne diseases. Diarrhoea is a major cause of the death of more than 2 million people per year worldwide, mostly children under the age of five. In fact, each day, nearly 1,000 children die due to preventable water and sanitation-related diarrhoeal diseases [6, 7].

Good quality water is a vital deciding factor for human health, which directly relates to the socioeconomic progress of a country [8–10]. Thus, the importance of water cannot be overemphasized. In fact, sustainable socioeconomic development and productive livelihoods can be built on access to water. Productive uses of water are employed at the household levels, including a range of small-scale domestic activities, in agriculture and industrial purposes. Although the quality requirement for water varies from one sector to the other and from one form of usage to the other, many of these activities have implications for public health, because it can result in the transmission of waterborne diseases [11–13].

These water-related infections are a result of the pollution of the water sources, due to anthropogenic influences, population growth, and urbanization [14]. The pollution thus leads to the development and propagation of pathogenic waterborne microorganisms. *Escherichia coli* is one of the major pathogens associated with waterborne diseases. Naturally, *Escherichia coli* (*E. coli*) is a facultative anaerobic bacterium that inhabits the large gastrointestinal tracts of warm-blooded animals and is a major normal flora associated with the human colon [15–17].

Therefore, the existence of *E. coli* in food or water normally signals recent faecal contamination or poor hygienic conditions in food processing facilities [18]. Thus, faecal contamination, poor sanitation measures, and poor storage conditions have a major influence on *E. coli* population [19, 20]. The mere existence of *E. coli* in water does not necessarily imply the presence of disease-causing microbes. However, it gives an indication of the possible existence of faecal-borne microorganisms such as Salmonella and hepatitis A [21, 22]. This accounts for the usage of *E. coli* as an indicator microbe to examine food and water samples aimed at detecting the levels of faecal contaminations [21].

Water safety management depends primarily on hazard identification through drinking water surveillance, which is “the continuous and vigilant public health assessment and review of the safety and acceptability of drinking water supplies” [23, 24]. This surveillance is critical to public health and safety, as it contributes and supports improvements in water quality and accessibility. The present study evaluated the health risks associated with sources of drinking water in the Dangme district in Ghana using *E. coli* as a bacteria source tracking tool measurement tool, aimed at ascertaining the paths leading to contamination of the water sources.

## 2. Materials and Methods

### 2.1. Study Area

**Figure 1:**
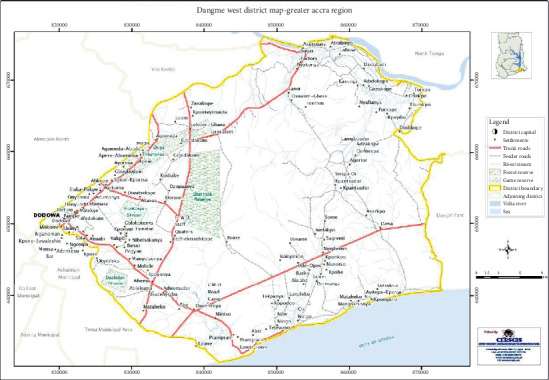
A map of the Dangme district of Ghana.

The Dangme West District (Figure 1) is situated in the southeastern part of Ghana, lying between longitude 5° 45′ south and 6° 05′ north and longitude 0° 05′ east and 0° 20′ west. The district has a total land area of 1,442 square kilometers, making it the largest in the Greater Accra Region. The land size represents 41.5% of the regional land area. The district forms part of sixteen (16) metropolitan, municipal, and districts in the Greater Accra Region of Ghana. The district has a 37 km Coastline and a 17 km stretch of the Volta River [25].

### 2.2. Topography and Drainage

The district forms the central portions of the Accra plains. The relief is generally gentle and undulating, a low plain with heights not exceeding 70 metres. The plains are punctuated in isolated areas by a few prominent inselbergs, isolated hills, outliers, and knolls scattered erratically over the area. Prominent relief features include the Yongua inselberg (427 metres) which appears conical in the air with a number of outliers close to the north of the district around Asutsuare and Osuwem areas, the Krabote inselberg also to the north and the Shai hills (289 metres) found towards the western portions of the district [26]. The mean annual rainfall increases from 762.5 milliliters on the coast to 1220 milliliters to the North and Northeast close to the foothills of Akwapim range and on the summit [26].

### 2.3. Climate and Vegetation

The southeastern coastal plain of Ghana, which encompasses the Dangme West District, is one of the hottest and driest parts of the country. Temperatures are, however, subjected to occasional and minimal moderating influences along the coast and altitudinal influences affected by the Akwapim range in the northwest. Temperatures are appreciably high for most parts of the year, with the highest during the main dry season (November–March) and the lowest during the short dry season (July–August). They average a few degrees lower on the coast and close to the Akwapim range than they do over most of the plains. The absolute maximum temperature is 40°C [26]. Along some stream courses, however, higher vegetation type ranging from thickets to light forest is common. Some light forest with tall trees is also found along the foothills of the Akwapim Ridge, especially around Dodowa, Ayikuma, and Agomeda areas. There is a Forest, Game, and Wildlife Reserve around the Shai hills [26].

### 2.4. Water Sources and Sanitation

The supply of potable water in the district is woefully inadequate, and only a few sections of the district have a regular supply of pipe-borne water. Analysis of the current water and sanitation in the district shows that more effort is needed to meet the 85% water and sanitation coverage. On the basis of the National Community Water and Sanitation Standards of 600 people per standpipe, 350 persons per borehole, and 150 persons per hand-dug well, the district has achieved about 66% coverage with 34% of the population lacking access to a potable water supply. As it stands now, below 37% of the district population has access to pipe-borne water. The remaining population in the district depends on untreated water sources. Visually, the water from the streams and dams are light brownish yellow caused by mostly decayed dead leaves. If turbulently disturbed, it turns to deep brownish yellow and some suspended soils can be seen [26].

### 2.5. Sample Collection and Analysis

Four hundred sixty-four (464) water samples were obtained from the drinking sources at the study location for analysis. Sampling duration was a year, and sampling was carried out throughout the wet and dry seasons in Ghana. The water samples were obtained from the surface (dams, river, canals, and streams) and underground (hand-dug wells and boreholes) water sources.

Sampling and care of samples were done following guidelines of the WHO [27] while analyses of samples were carried out as described by APHA [28, 29].

Sterile bottles were used to collect the water samples from each of the sites. Samples were taken to the laboratory for analyses. The most probable number method (MPN) illustrated by Prescott et al. [30] was used to determine *E. coli* counts. A total of 15 tubes comprising 5 tubes for each of three dilution factors (0.1 ml, 1 ml, and 10 ml) were used for inoculation in the study. As a result, three sets, each containing five tubes, were used in the presumptive test. In the first set, each of the five tubes holding 10 ml of MacConkey Broth with double strength was inoculated with 10 ml of the water sample. The tubes in the second set, which contain 10 ml of single strength MAC broth, were inoculated with 1 ml of subwater samples and the remaining five tubes containing 10 ml of single strength MAC broth were inoculated with 0.1 ml of the sample. After that, the tubes were incubated at 37°C for 24 hours for total coliforms and 44°C for faecal coliforms. The tubes were then examined visually for turbidity. Each tube contains an inverted Durham tube (this is a very small test-tube), which is examined for gas production (bubble) and the media is examined for acid production, i.e, change in colour from pink to yellow.

Confirmation of *E. coli* was carried out by aseptically plating from tubes with acid and gas formation onto L-EMB agar in Petri dishes to obtain pure isolates. The plates were incubated at 35°C with a standard error of 0.5°C for 18–24 hours. The incubated plates were examined for centrally dark and flat colonies having or not having a metallic sheen. These morphological features are associated with *E. coli*. A maximum of 5 colonies from each L-EMB plate was inoculated onto slants of PCA and incubated at 35°C for about 24 hours to prepare stork culture. API20E was used to identify a 24 hr culture of each purified isolate following the manufacturer's instructions.

## 3. Results

Table 1 shows the range of seasonal *E. coli* counts (MPN/100 ml) in water. *E. coli* populations in samples sourced from hand-dug wells, boreholes, and stream all had a zero-minimum count across both seasons. There was a low count of *E. coli* in water samples obtained from river sources in the wet season. The range of *E. coli* counts (1.2 × 10^1^–7.9 × 10^1^ MPN/100 ml) was observed in samples collected from dams throughout the wet season.

A summary of comparative analysis between *E. coli* counts measured in MPN/100 ml in samples from the water sources is presented in Table 2. The highest counts for *E. coli* (59.67 ± 45.08) were identified in water samples collected from the canal sources in the wet season. On the other hand, the lowest counts (0–0.1 × 101 MNP/100 ml) were identified in the dry season from samples sourced from the rivers.

Analysis of mean variations and confidence intervals for *E. coli* proportions throughout the wet and dry seasons is shown in Tables 3 and 4, respectively. There was a significant difference between mean proportions of *E. coli* from boreholes and canal water sources in the wet season. Mean differences of counts among boreholes, river, and canals in the wet season were also significant.

**Figure 2:**
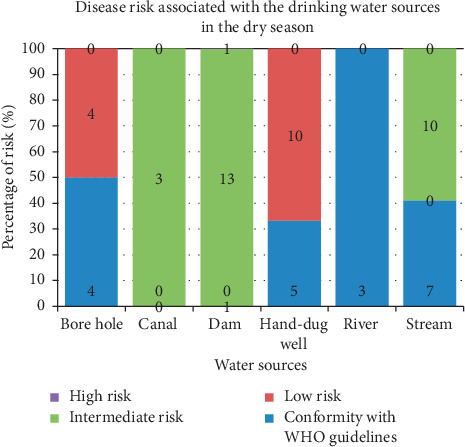
WHO disease risk levels of water sources in the wet season.

Findings of the investigation of the extent of disease risk associated with the drinking water sources in both seasons (wet and dry) are shown in Figures 1 and 2, respectively. This analysis was carried using WHO drinking water risk assessment guidelines. According to the World Health Organization, a zero count of *E. coli* per 100 ml of water is considered safe for drinking. A count of 1–10 MPN/100 ml is regarded as low risk; 11–100 MPN/100 ml is medium risk. Finally, an *E. coli* count greater than 100 MPN/100 ml is adjudged high risk.

With reference to Figure 2, it is evident that the dam and canal water sources revealed intermediate disease risk (100%) throughout the wet season. All water samples (100%) sourced from the canals showed an intermediate risk level followed by that of a dam with an 87% intermediate risk level. An intermediate risk level of 59% was observed in water obtained from streams.

Water from rivers had 100% compliance with WHO standards. However, water from canals and dams showed no conformity with the guidelines, thereby implying the substantial health risks they pose to human health. Water sources from hand-dug wells revealed a 67% low-risk level. Sources from boreholes showed 50% low risk and 50% conformity with standards set by the WHO. Stream water sources had a 59% intermediate risk level with 41% conformity with the WHO guidelines.

**Figure 3:**
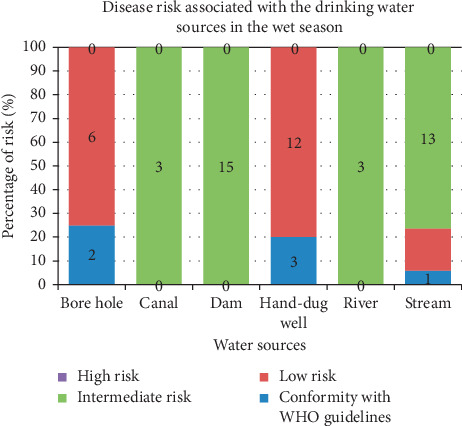
WHO disease risk levels of water sources in the dry season.

The extent of disease risk of the various water sources during the dry season is presented in Figure 3. Data from Figure 3 show an important trend. All of the sources did not show a high-risk level in the dry season, just as observed in the wet season.

All water sources from rivers, hand-dug wells, and canals had 100% intermediate risk. Boreholes had the lowest disease risk, with a 75% low-risk level and 25% compliance with WHO standards. This was followed by hand-dug wells at 80% low-risk level. On the other hand, dams showed a high disease risk.

## 4. Discussion

The present study evaluated the health risks associated with sources of drinking water in the Dangme West District in Ghana using *E. coli* as a measurement tool, aimed at ascertaining the paths leading to contamination of the water sources. *E. coli* is best suited as an indicator for faecal coliforms because there are fewer instances of encountering false positives [31, 32]. Also, *E. coli* enumerations are better indicators of faecal pollution than faecal coliform counts [33]. The reason being that certain strains of faecal coliform bacteria are capable of multiplying in the environs which can lead to pseudoelevation of indicator levels [34]. Additionally, recent studies have identified a direct association between the density of *E. coli* microbes in water and the prevalence of water-related gastroenteritis [35]. This, among other reasons, accounts for the use of *E. coli* counts as an indicator of water quality and, by extension, a signal of human, domestic, and natural sources of faecal contamination [18, 36–39].

In this study, we found *E. coli* counts to be predominantly higher in the wet season than the dry season (Table 1). Terrestrial wastes usually budge into most water sources in periods of excessive hailstorms or downpours, which could cause higher levels of bacteria counts during wet seasons than dry seasons [40, 41]. This could account for the higher counts observed in the water samples collected during the wet season than in the dry season. Although there were higher counts in the wet season than in the dry season, several reasons could also explain the counts observed in the dry season. First of all, water shortage and brisk multiplication of bacteria as a result of suitable conditions such as temperature throughout the dry season [42] could account for such a revelation. It is worth noting, rainfalls provided higher water volumes and large surface area for rapid microbial growth, leading to higher microbial counts and subsequently, higher extents of pollution in the wet season. Furthermore, bacterial pollutants in dumpsites, human waste, and heating systems could be drained into the different water sources during the wet season, after which rapid multiplication occurs. These sources are thus, probable sources of transmitting pathogens resulting in more critical health problems in rural neighborhoods.

Findings of the investigation of the extent of disease risk associated with the drinking water sources in both seasons across the seasons (Figures 1 and 2) using WHO drinking water risk assessment guidelines. Water samples from the canals showed an intermediate risk level. An intermediate risk level of 59% was also observed in water obtained from streams. However, in the dry season, none of the water sources showed a high-risk level invariance to observations made in the wet season. This observation is consistent with several similar studies done in the developing world [43].

Undisputedly, anthropogenic activities and faecal releases as preeminent contributing determinants of faecal pollution could explain the high enumerations of *E. coli* in samples collected from various water sources. However, since the comparative significance of precise animals with regard to *E. coli* counts were not befittingly evaluated, it is difficult to confidently determine their contribution to the higher counts observed. Possibly, the higher counts could be associated with other related factors including animal populace density and exploitation of areas close to the water sampling sources or sites. Though the current study did not reveal anthropogenic input as a critical cause of the presence of high faecal coliforms in most water samples investigated, these results hint that human faecal contaminations could be camouflaged by other sources (such as wild and domestic animals) especially when high counts are observed.

It is important to note that a number of observations were made concerning the environmental conditions in the district during the research period, which gives insights of the pollution of the water sources. First, there were limited dumpsites for waste disposal in the district, which could account for the use of streams as alternative waste dumping sites. Moreover, it was observed that inhabitants in some communities in the district adopt unhygienic activities particularly, defecation and urination in the open and in drain ways, which eventually gets into water bodies.

Second, we observed that their groundwater sources had similar characteristics, that is, the absence of efficient physical barriers such as concrete seals, well linings, hygienic covers, and secured aseptic lids among several others capable of avoiding terrestrial runoff comprising anthropogenic and animal waste which will assuredly pollute the sources. This could be linked to the detection of *E. coli* counts in the groundwater sources in the present study. In view of the essence of physical barriers in wells, WHO [44] purported that groundwater is less susceptible to pollution as a result of the presence of barriers. Hence, contamination levels will rise if these barriers are compromised. This is particularly worrisome because, according to Chapman [45] when groundwater is polluted, it can remain polluted for many years. The inference of this result is pivotal and, as such, cannot be overelaborated.

## 5. Conclusions

The present study showed that most water sources analyzed were contaminated with bacterial pathogens surpassing recommended standards thereby suggesting that residents living in the rural neighborhoods of the study area are exposed to heightened risks and susceptibility to waterborne diseases or health complications. A number of practices are associated with contamination of the water sources especially anthropogenic activities. Therefore, regular tests of water quality in rural vicinities are not only advantageous but necessary.

Therefore, the government, in close collaboration with community chiefs and elders of the district, should develop a keen interest in monitoring water quality in those environments. Government and other stakeholders must also initiate education on environmental awareness including hygienic practices. Additionally, education on proper water storage practices, sanitary ways of handling water, among others, should be done to help to limit water pollution for safe consumption.

## Figures and Tables

**Table 1:** The range of seasonal *E. coli* counts (MPN/100 ml) in water.

Water sources	Seasonal counts (MPN/100 ml)	
	Dry season	Wet season
Borehole	0–0.2 × 10^1^	0–0.2 × 10^1^
Canal	2.3 × 10^1^–1.1 × 10^2^	3.1 × 10^1^–6.3 × 10^1^
Dam	0.7 × 10^1^–1.1 × 10^2^	1.2 × 10^1^–7.9 × 10^1^
Hand-dug well	0–0.2 × 10^1^	0–0.4 × 10^1^
River	0–0.1 × 10^1^	0.2 × 10^1^–0.7 × 10^1^
Streams	0–3.3 × 10^1^	0–3.3 × 10^1^

**Table 2:** Comparison of *E. coli* counts (MPN/100 ml) in the various water sources (SD = standard deviation, df = degree of freedom, Min = minimum, and Max = maximum).

Water sources	Dry season				Wet season				*P* value
	Mean ± SD	Min	Max	df	Mean ± SD	Min	Max	df	
Bore hole	0.63 ± 0.74	0	2	7	1 ± 0.76	0	2	7	0.090
Canal	47.67 ± 16.04	31	63	2	59.67 ± 45.08	23	110	2	0.280
Dam	38.07 ± 30.71	7	110	14	39.87 ± 19.76	12	79	14	0.350
Hand-dug well	0.93 ± 0.80	0	2	14	1.47 ± 1.06	0	4	14	0.001
River	0 ± 0	0	0	2	4.33 ± 2.52	2	7	2	0.050
Stream	15.06 ± 13.59	0	33	16	20.77 ± 11.05	0	31	16	0.020

**Table 3:** Analysis of mean differences and confidence intervals for *E. coli* levels in the dry season (test value = 6; the mean difference is significant at *P* ≤ 0.05).

Water sources	*T*	df	Sis (2-tailed)	Mean difference	95% confidence interval	
					Upper limit	Lower limit
Borehole	2.376	7	0.049	0.625	0.00	1.25
Canal	5.147	2	0.036	47.667	7.82	87.52
Dam	4.800	14	0.001	38.067	21.06	55.08
Hand-dug well	4.525	14	0.001	0.933	0.49	1.38
River	—	2	—	—	—	—
Stream	4.569	16	0.001	15.059	8.07	22.05

**Table 4:** Analysis of mean differences and confidence intervals for *E. coli* levels in the wet season (test value = 6; the mean difference is significant at *P* ≤ 0.05).

Water sources	*T*	df	Sis (2-tailed)	Mean difference	95% confidence interval	
					Upper limit	Lower limit
Bore hole	3.742	7	0.007	1.000	0.37	1.63
Canal	2.292	2	0.149	59.667	−52.32	171.66
Dam	7.814	14	0.001	39.867	28.92	50.81
Hand-dug well	5.358	14	0.001	1.467	0.88	2.05
River	2.982	2	0.096	4.333	−1.92	10.58
Stream	7.745	16	0.001	20.765	15.08	26.45

## Data Availability

The data used to support the findings of this study are included within the article.
